# The dynamic functional connectivity fingerprint of high-grade gliomas

**DOI:** 10.1038/s41598-023-37478-2

**Published:** 2023-06-27

**Authors:** Manuela Moretto, Erica Silvestri, Silvia Facchini, Mariagiulia Anglani, Diego Cecchin, Maurizio Corbetta, Alessandra Bertoldo

**Affiliations:** 1 Padova Neuroscience Center, University of Padova, 35131 Padova, Italy; 2 Department of Information Engineering, University of Padova, Via G. Gradenigo 6/B, 35131 Padova, Italy; 3 Department of Neuroscience, University of Padova, 35121 Padova, Italy; 4 Neuroradiology Unit, University of Padova, 35121 Padova, Italy; 5 Unit of Nuclear Medicine, University of Padova, 35121 Padova, Italy; 6 Venetian Institute of Molecular Medicine, 35131 Padova, Italy

**Keywords:** Neuroscience, Tumour biomarkers

## Abstract

Resting state fMRI has been used in many studies to investigate the impact of brain tumours on functional connectivity (FC). However, these studies have so far assumed that FC is stationary, disregarding the fact that the brain fluctuates over dynamic states. Here we utilised resting state fMRI data from 33 patients with high-grade gliomas and 33 healthy controls to examine the dynamic interplay between resting-state networks and to gain insights into the impact of brain tumours on functional dynamics. By employing Hidden Markov Models, we demonstrated that functional dynamics persist even in the presence of a high-grade glioma, and that patients exhibited a global decrease of connections strength, as well as of network segregation. Furthermore, through a multivariate analysis, we demonstrated that patients’ cognitive scores are highly predictive of pathological dynamics, thus supporting our hypothesis that functional dynamics could serve as valuable biomarkers for better understanding the traits of high-grade gliomas.

## Introduction

Gliomas are the most common malignant primary brain tumours in adults, with an annual incidence of 4.7–5.7 new cases per 100,000 individuals^1^. They are associated with significant morbidity and mortality^2^. The most aggressive form, glioblastoma, represents a lethal tumour with fewer than 5% of patients surviving at 5 years despite optimal treatment^1^. The most effective treatment of gliomas is neurosurgical resection^3^ followed by radio and/or chemotherapy^4^. Many studies have proven evidence that overall survival can be improved with gross total resection, as opposed to subtotal resection, for both high-grade gliomas (HGG) and low-grade gliomas (LGG)^5–7^. However, extensive resection may have an impact on cognitive functions, worsening them and leading to permanent neurological dysfunctions. Importantly, presurgical cognitive status has been shown to be a strong predictor of patients’ overall survival^8,9^, highlighting the significance of cognitive functions assessment for decision‐making throughout the clinical care and treatment process. Therefore, it is crucial to study how functional networks are distributed in the presurgical brain, as an increasing number of studies have demonstrated that cognitive deficits in tumour patients go beyond local effects and instead depend on the organisation of functional networks at rest^10–12^.

To date, there have been very few studies investigating potential alterations in resting-state functional connectivity (rs-FC) within and between resting-state networks (RSNs) in patients with HGG (refer to^13^ for a comprehensive review). While most of these studies have focused on specific RSNs, such as the language network (LANG)^14–16^, the default-mode network (DMN)^12,17–21^ and the fronto-parietal network (FPN)^16–18^, only a limited number of papers have examined these alterations at the whole-brain level^19,22–27^. In studies investigating the LANG, an overall decrease of rs-FC^14,15^ as well as network strength^16^ in patients compared to healthy controls has been reported. In contrast, regarding the DMN, the findings are much more inconsistent across studies. Some studies have reported an increase in overall rs-FC within the network^18^, while others have shown a decrease^12^. Similarly, the findings for the FPN are ambiguous: one study^18^ reported an increase in rs-FC among the posterior cingulate cortex, precuneus, and the frontal cortex or within the contralateral hemisphere in patients compared to healthy controls, while another study^21^ observed a global reduction in FPN strength. Although these studies provide a better characterisation of the functional status of various networks, it is important to note that they all rely on static analyses, disregarding the dynamic rearrangement of RSNs that occurs even during resting conditions^28^. However, for a comprehensive characterisation of a patient's brain functional connectivity, it is essential to consider the dynamic behaviour of RSNs. An approach that explicitly models the dynamics of the BOLD signal in networks of interest is the Hidden Markov Model (HMM)^29^. This approach decomposes the time-varying BOLD signal from predefined regions or networks into a sequence of discrete brain states that recur over time. Each brain state is characterised by a specific FC profile and a spatiotemporal pattern of BOLD activation. By estimating the states and the likely sequence of transitions between them, two summary metrics can be derived for each individual: the frequency of transitions between states, known as the switching rate (SR), and the overall proportion of time that an individual spends in each brain state, known as the state's fractional occupancy (FO).

In recent years, the HMM has been utilised to assess dynamic FC in both healthy individuals^29–31^ and those with brain disorders^32–34^. These studies have provided evidence that dynamic descriptors of brain states accurately predict symptom severity in clinical populations, such as schizophrenia^34^, or are strongly correlated with medications in patients with multiple sclerosis^32^.

Despite these findings, to the best of our knowledge, there is still a lack of studies investigating functional dynamics in brain tumours in the existing literature.

In this study, we employ a framework to track the dynamics of rs-FC in brain tumour patients, utilising resting-state functional Magnetic Resonance Imaging (rs-fMRI) data from patients with HGG and healthy controls (HCs). Specifically, we aim to address three research questions: (1) Do brain functional dynamics exist even in the presence of an HGG? (2) How do the patterns of dynamic rs-FC in patients differ from those of HCs? (3) If there are modifications in the patients’ dynamics, could they be related to pathophysiological features such as lesion extension or cognitive status?

## Results

To investigate modifications in the brain’s functional dynamics in patients with HGG, we utilised the statistical model-based method of HMM. This method employs temporally concatenated rs-fMRI time courses to obtain a probabilistic description of the time-varying neural processes in the form of a sequence of discrete brain states.

In this study, we utilised rs-fMRI data from 33 patients with HGG, acquired at the University Hospital of Padova. Additionally, we used rs-fMRI data from 33 HCs sourced from the publicly available MPI-Leipzig Mind-Brain-Body dataset^35^. Within the HMM framework, the temporally concatenated BOLD time courses of 45 independent components (ICs), which represent spatially independent co-activated brain areas, were grouped into 10 RSNs. These ICs were then described using a repertoire of 6 brain states, each characterised by a specific FC profile that denotes the level of connectivity between ICs.

After obtaining the sequence of brain states visited by each subject at each time point during the entire acquisition, the transition probabilities among states were computed. To determine whether brain functional dynamics exist even in the presence of HGG, the frequency of transitions between states (i.e., the switching rate) and the proportion of time spent in each state (i.e., the fractional occupancy) were compared between patients and healthy controls. Next, to investigate potential modifications in the patterns of dynamic connectivity between pathological and healthy states, both a gradient-based and a graph-based analysis were performed on the FC matrices associated with each state. Finally, the relationship between the occupancy in a state and the cognitive status of a patient was investigated using a multivariate analysis, where neuropsychological test scores were used as predictors.

### Patient cohort

The patient cohort comprised 33 patients (15 female, mean age 60.5 ± 12.9y) affected by left-HGG (N = 18), right-HGG (N = 11) or bilateral-HGG (N = 4). Of the total cohort, 26 patients had wild-type glioblastomas, while 3 patients had the IDH1 mutation. Supplementary Table 1 provides the demographic and clinical information at the individual level.

The distribution of tumour lesions in our dataset was sparse, primarily involving association regions with high functional connectivity, such as the right frontal and left temporal lobes. The maximum overlap value among patients was 27.3% (Supplementary Fig. 1), which aligns with the expected frequency distribution reported by Mandal and colleagues^36^.

### Functional dynamics are not destroyed in HGG patients

The HMM approach returned 6 brain states to which participants transitioned during the rs-fMRI scan. These states, denoted as S1, S2, S3, S4, S5, and S6, were characterised by distinct FC matrices and spatiotemporal patterns of BOLD activation.

To investigate possible alterations in functional dynamics between patients and HCs, we initially derived two temporal metrics, namely the SR and FO, at the single-subject level.

**Figure 1:**
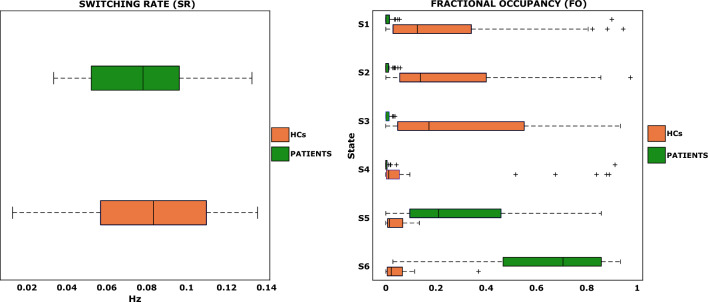
Left panel reports the boxplots of the frequency of transition between states (switching rate, SR) and right panel reports the fractional occupancy (FO), ranging from 0 to 1, in each state (y-axis) and for the two groups of subjects separately (HCs in orange and patients in green).

Regarding the frequency of transitions between states (SR), no statistically significant differences were found between patients (mean SR = 0.076 Hz, corresponding to a transition every 12.8 s) and HCs (mean SR = 0.08 Hz, corresponding to a transition every 12.5 s). This suggests that, despite the presence of the lesion, brain dynamics are preserved, albeit slowed down. Although the SR was not sufficient to discriminate between the two groups, the advantage of using HMM was its ability to differentiate patients from HCs based on the FO metric, which represents the proportion of time spent by each individual in a particular state. In Fig. 1, the FO distribution for each of the 6 brain states is reported separately for patients and controls. A paired Wilcoxon’s test followed by False Discovery Rate correction (α = 0.01) was conducted to test for differences in FO between the two groups. The statistical test revealed significant differences between patients and HCs in all 6 states (all p-values < 5e-5). Consequently, a state was labelled as “pathological state” if it was predominantly occupied by patients, while states predominantly occupied by HCs were labelled as “healthy states”. On average, HCs spent 25% of the total acquisition time in S1 and S2, 29% in S3, and 13% in S4. In contrast, patients spent an average of 30% of time in S5 and 62% in S6. Specifically, the FO index allowed us to further separate patients into two subgroups: those who mostly occupied S5 and those who predominantly occupied S6. Among the 33 patients, 8 were found to have the highest occupancy in S5, while 23 mostly occupied S6. A strong and expected anticorrelation (Pearson’s R = 0.99, p-value < 2e−25) between patients’ occupancy in S5 and S6 was also reported. This highlights the dichotomous behaviour of these two pathological states and emphasises the importance of characterising both to understand the functional dynamics of each patient.

In addition to SR and FO, the model allows the quantification of the transition probabilities (as shown in Supplementary Fig. 2), which assess the likelihood of transitions between states throughout the entire acquisition at the individual level. Focusing on the two subgroups of patients, we found that when a patient falls into S5, there is a high probability of transitioning to S6 (probability = 0.41), while the reverse path, from S6 to S5, is less likely (probability = 0.20). Furthermore, on average, the SR of the 8 patients who mostly occupied S5 was higher (mean SR = 0.1 Hz, corresponding to a transition every 10 s) than that of the 23 patients who mostly occupied S6 (mean SR = 0.07 Hz, corresponding to a transition every 13.8 s).

### Weaker connectivity strength in HGG patients

**Figure 2:**
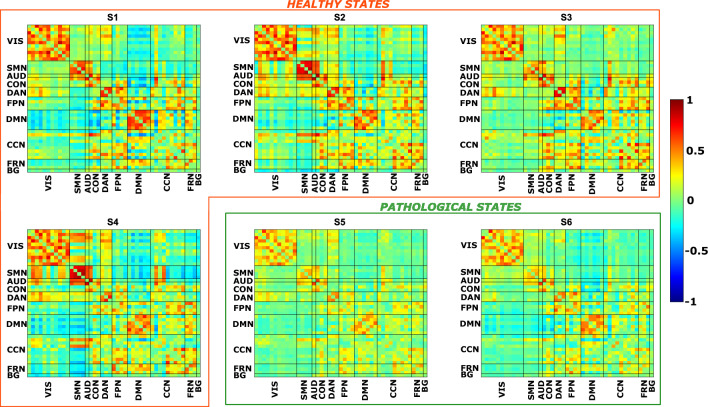
Each panel represents the functional connectivity (FC) matrix associated with a particular brain state (S1, S2, etc.). In both the x- and y-axis, we have the 45 independent components (ICs), divided in the 10 RSNs. Warm colours represent high positive correlations values between ICs, whereas cool colours represent high anti-correlations values. We grouped together healthy states (orange box) and pathological states (green box). VIS = visual; SMN = sensorimotor; AUD = auditory; CON = cingulo-opercularis; DAN = dorsal-attention; FPN = fronto-parietal; DMN = default-mode; CCN = cognitive-control; FRN = frontal; BG = basal ganglia.

Besides individual metrics of brain dynamics, the HMM approach also provided functional correlation matrices associated with each brain state. These matrices were estimated at the population level and provide average information on the functional connectivity between two ICs when a specific state is active. In Fig. 2, we present the FC maps of each state and highlight whether they correspond to a healthy or pathological condition. After sparsification of these matrices, we found a statistically significant decrease of the strength of connections, represented by the connectivity value, in the two pathological states, S5 and S6, compared to all the healthy states (Wilcoxon’s test, α = 0.05, FDR corrected).

### Networks organisation captured by the principal gradient is preserved across states

**Figure 3:**
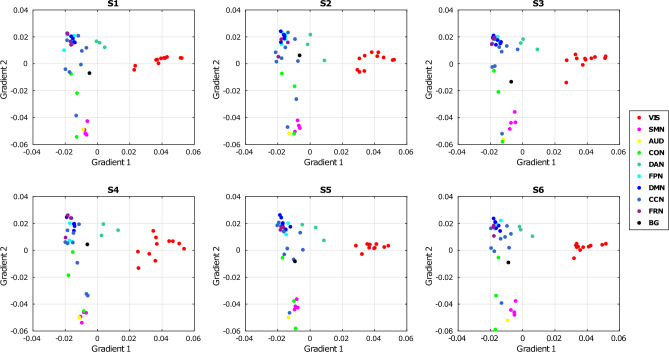
shows the plot of the 1st gradient VS 2nd gradient for each brain state. Each point represents one of the 45 ICs and different colours depict different RSNs. The 1st gradient is preserved across states: the organisation of RSNs is preserved across states. VIS = visual; SMN = sensorimotor; AUD = auditory; CON = cingulo-opercularis; DAN = dorsal-attention; FPN = fronto-parietal; DMN = default-mode; CCN = cognitive-control; FRN = frontal; BG = basal ganglia.

Considering that pathological states showed a weaker strength of connections compared to healthy states, we performed a gradient analysis^37^ to investigate whether this decrease could be attributed to a modification in the organisation of the underlying networks. In Fig. 3, we present the values of the 1st gradient plotted against those of the 2nd gradient for each IC assigned to one of the 10 RSNs and for each brain state. Across all 6 states, the principal gradient, which accounts for the highest variance in FC, is anchored at one end by visual components (commonly referred as unimodal areas^37^) and at the other end by components belonging to higher-order networks (such as the DMN, FPN and cognitive control network-CCN, often referred to as transmodal areas^37^). The cosine similarity computed between the principal gradients of the states ranged between 0.98 and 0.99, indicating a preservation of networks organisation across all states, regardless of whether they were healthy or pathological.

### Decrease of networks segregation in pathological states

**Figure 4:**
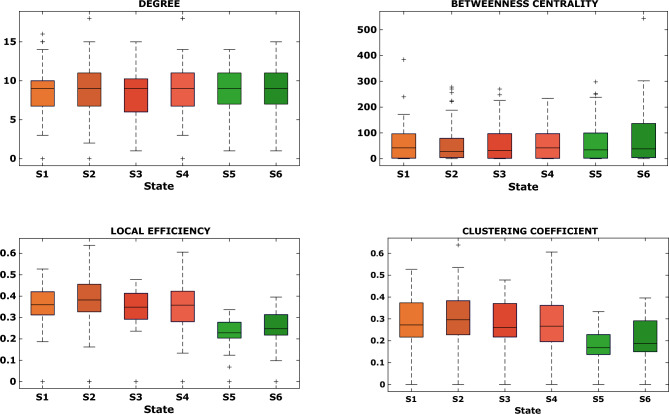
The two top panels report the distributions of the two metrics of centrality (degree and betweenness centrality), in the form of boxplot, evaluated on the sparsified FC matrices of each brain state (x-axis). In the bottom panels, the distributions of the two metrics of network segregation (local efficiency and clustering coefficient) are reported.

Once it was verified that the 6 states shared a common network organisation, we conducted a graph-based analysis to examine the role of ICs within each FC matrix associated with each brain state. This analysis focused on metrics of centrality, segregation and integration of the networks. Degree and betweenness centrality were used as measures of centrality, while local efficiency and clustering coefficient were used as measures of segregation, and global efficiency was used as measure of integration. Figure 4 displays the distributions of centrality and segregation metrics for all 6 brain states. A paired Wilcoxon’s test followed by FDR correction (α = 0.01) was employed to test for differences in the other four graph metrics between pairs of states. The statistical test revealed statistically significant differences between patients and HCs in all 6 states (all p-values < 5e−5). Degree and betweenness centrality did not show any differences between states (all p-values > 0.6). However, when quantifying network segregation, we observed statistically significant differences in both pathological states, S5 and S6, compared to all healthy states in terms of local efficiency (all p-values < 1e−4) and clustering coefficient (all p-values < 0.005). Additionally, we observed a decrease in global efficiency for the two pathological states (0.15 for S5, 0.16 for S6), compared to the healthy states (0.21 for S5, 0.23 for S2, 0.22 for S3, 0.22 for S4). This decrease in segregation and integration metrics suggests that networks exchange information less efficiently in pathological states.

**Figure 5:**
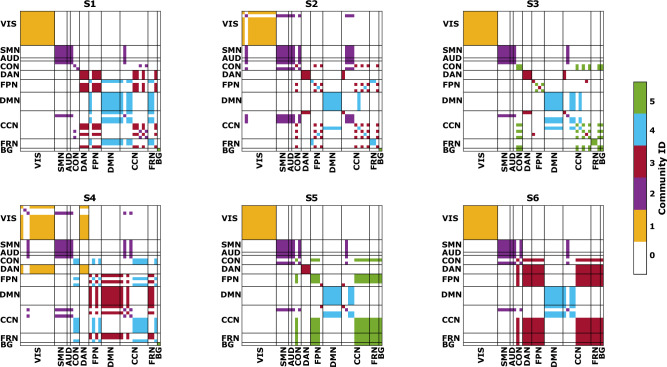
Each figure represents the modular organisation of a brain state (S1, S2, etc.) obtained as explained in the main text. Different colours are representative of a distinct community. In both the x- and y-axis, we have the 45 ICs, divided in the 10 RSNs. VIS = visual; SMN = sensorimotor; AUD = auditory; CON = cingulo-opercularis; DAN = dorsal-attention; FPN = fronto-parietal; DMN = default-mode; CCN = cognitive-control; FRN = frontal; BG = basal ganglia.

Furthermore, we computed the Louvain’s modularity to detect communities within the states (Fig. 5). On average, the modularity was 0.55 for S1, 0.49 for S2, 0.50 for S3, 0.48 for S4, 0.54 for S5 and 0.58 for S6. A modularity of 0 indicates the presence of only one functional community, while a modularity of 1 indicates as many communities as the number of ICs. In S6, only four communities were detected, while all other states had five communities. The two pathological states exhibited a similar pattern of integration and segregation of RSNs, except for the dorsal attention network (DAN), which was completely segregated in S5 but highly integrated with other higher-order networks in S6. Among the Jaccard similarity values computed between the modular matrices of the states shown in Fig. 5, the highest coefficient of 0.77 was found between the two pathological states, confirming a similar arrangement of functional modules within these two states. The other similarity values are reported in Supplementary Table 2.

### The dynamic stratification of patients depends on their cognitive performances

**Figure 6:**
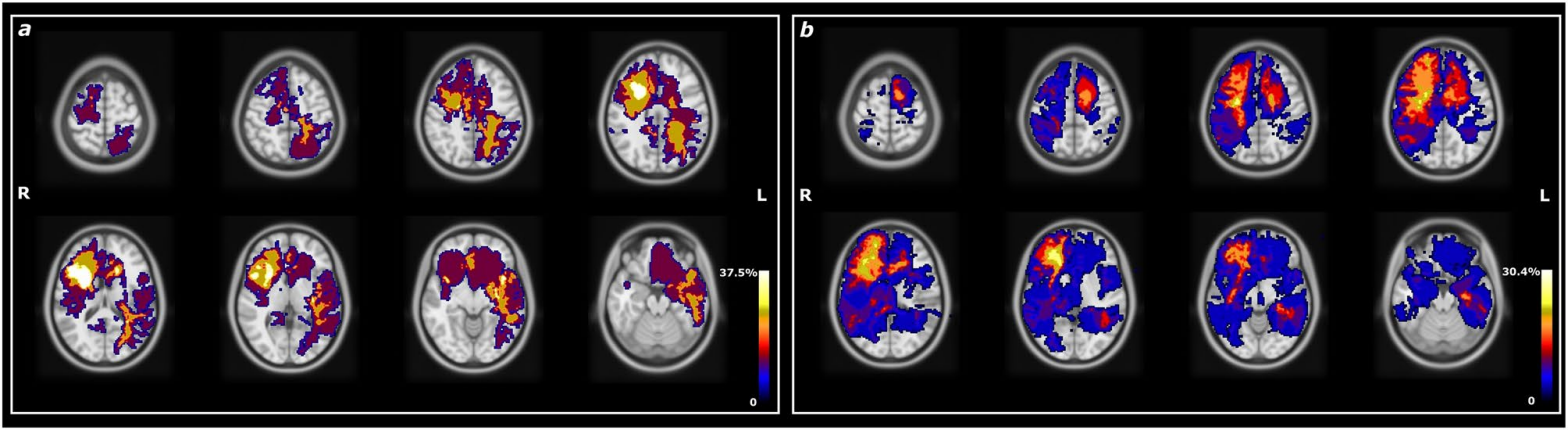
Lesion frequency map of the 8 patients who mostly persist in S5 (**a**) and of the 23 patients who mostly persist in S6 (**b**). Maps are over-imposed on the MNI atlas (grey scale). Radiological convention.

In the previous paragraphs, we showed how HMM could effectively classify patients in one of the two pathological states. To determine whether this classification into S5 or S6 was influenced by pathophysiological features related to the lesion, such as the extension or location of the lesion, we initially computed Pearson’s correlation between the lesion extension and the maximal FO of each patient in S5 or S6. The occupancy of patients in S5 or S6 was found to be independent of the lesion extension. Pearson's correlation coefficient between the patient’s lesion extension and FO in S5 was 0.31 (p-value = 0.07), while the correlation with FO in S6 was 0.04 (p-value = 0.8). In addition, lesion location was not associated with the persistence in a particular state. Among the 8 patients who mostly persisted in S5, 2 had right-hemisphere glioma, 4 had left-hemisphere glioma and 2 had bilateral glioma. Figure 6 shows the lesion frequency map of the 8 patients who predominantly persisted in S5 (panel A) and the lesion frequency map of the 23 patients who predominantly persisted in S6 (panel B). Overall, no significant relationships were found between lesion characteristics and the FO in either of the two pathological states.

Subsequently, we explored the potential relationship between occupancy and the cognitive abilities of patients, which were assessed using a battery of 24 neuropsychological tests that covered various cognitive domains (memory, attention, executive functions, language, visuo-spatial functions). Z-scores values for these tests are reported in Table 1.

**Table 1:** Scores of neuropsychological tests after normalisation. OCS = Oxford Cognitive Screen.

Test	Z-Score Median ± MAD	Range [Min Max]
OCS denomination	0.67 ± 1.22	[− 4.33 0.67]
OCS sentence reading	0.33 ± 1.67	[− 8 0.33]
OCS number writing	0 ± 1.07	[− 5 0]
OCS calculation	0.4 ± 0.93	[− 3.6 0.4]
OCS hearths overall accuracy	− 0.03 ± 0.63	[− 2.78 0.73]
OCS egocentric neglect	− 0.07 ± 0.81	[− 3.4 1.93]
OCS imitating gesture right	0.5 ± 0.64	[− 3.67 0.5]
OCS imitating gesture left	0.5 ± 1.27	[− 9.5 0.5]
OCS verbal memory	0.75 ± 1.17	[− 4.25 0.75]
OCS episodic memory	0.25 ± 0.92	[− 2.25 0.25]
Memint 10S	− 0.55 ± 1.07	[− 2.78 1.58]
Memint 30S	− 1.43 ± 1.30	[− 5.76 0.79]
Prose memory-immediate recall	− 0.91 ± 0.86	[− 2.39 2.19]
Prose memory-delayed recall	− 1.1 ± 1.13	[− 4.63 1.37]
Trail making test—A	0.43 ± 0.92	[− 4.67 1.15]
Trail making test—B	− 0.5 ± 1.76	[− 7.46 1.30]
Phonemic fluency	− 1.23 ± 0.89	[− 3.45 2.39]
Corsi forward	− 0.17 ± 1.02	[− 2.21 1.87]
Corsi backward	− 0.69 ± 1.21	[− 4.91 1.87]
Digit span-forward	− 0.97 ± 0.92	[− 6.27 2.5]
Digit span-backward	− 0.53 ± 1.26	[− 5.37 0.93]
Boston naming test-RS	− 0.8 ± 1.46	[− 8.30 0.8]
Nine-hole peg right hand	− 1.15 ± 1.69	[− 7.69 1.39]
Nine-hole peg left hand	− 1.48 ± 1.43	[− 7.29 1.53]

Initially, we conducted a univariate analysis (Wilcoxon’s test, α = 0.05), which revealed that only the test associated with egocentric neglect exhibited a significant difference between the two patient subgroups: those predominantly occupying S5 and those predominantly occupying S6 (p-value = 0.007).

**Figure 7:**
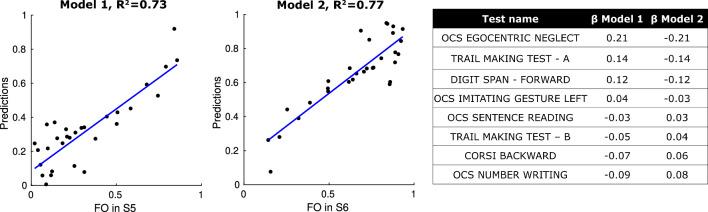
The left panels report the results of model 1 and model 2 through a scatterplot between the FO in S5 or in S6 (x-axis) and its prediction (y-axis). The right table reports, for both the models, the β weights associated with each of the 8 selected predictors.

Secondly, we moved to a multivariate analysis framework to predict the FO in either S5 or S6 using the complete set of cognitive tests as predictors. The hierarchical clustering approach, adopted for features reduction, identified 17 out 24 cognitive tests. Further reduction was performed through stepwise regression. The final set of tests used for predicting FO in S5 (model 1) or FO in S6 (model 2) consisted of the same 8 regressors: the normalised scores of the Oxford Cognitive Screen (OCS)_egocentric neglect, Trail Making Test—A, Digit Span—forward, OCS_Imitating gesture left, OCS_Sentence reading, Trail Making Test—B, Corsi backward, OCS_Number writing. Figure 7 presents the results of the multivariate analysis, demonstrating a strong correlation between the measured FO in S5 or S6 and their respective predictions. Model 1 achieved an R^2^ of 0.73, while model 2 achieved an R^2^ of 0.77.

As reported in the table of Fig. 7, the weights (β) associated with each predictor in both models exhibited similar amplitudes but opposite signs. The OCS score for egocentric neglect had the highest predictive value (β_model 1_ = 0.21, β_model 2_ = − 0.21), confirming the findings of the previous univariate analysis. In addition to this test, in model 1, the highest β values were associated with tests measuring simple attentive functions (Trail Making Test—A, Digit Span—forward). Conversely, in model 2, the highest β values were associated with tests involving higher cognitive load and requiring executive functioning (Trail Making Test—B, Corsi backward).

## Discussion

Using rs-fMRI data from 33 patients with high-grade gliomas and 33 healthy controls, we exploited the statistical framework of HMM applied to the BOLD time courses of 45 independent components belonging to 10 RSNs to investigate the evolution of brain functional dynamics in patients and how do they differ from healthy ones.

Our analysis revealed six optimal dynamic brain states in which brain activity reorganises during rest, representing recurring patterns of FC that repeat over time.

The frequency of transitions between brain states (i.e., the switching rate) fell within the range of meaningful frequencies observed in BOLD rs-fMRI data (< 0.1 Hz)^38^. Patients exhibited slightly slower SR compared to healthy controls, suggesting that functional dynamics persist even in the presence of tumours, albeit at a reduced pace. Indeed, when we looked at the probability of occurrence of brain states (i.e., the fractional occupancy), we found that HMM allowed clear differentiation between healthy and pathological states. Controls predominantly occupied states S1, S2, S3 and S4, while patients mainly occupied states S5 and S6, albeit to varying degrees. Notably, S6 emerged as the most representative pathological state, with 69.7% of patients spending the majority of their time in this state. Furthermore, we observed that the transition probability from the other pathological state, S5, to S6 was the highest, at 0.41, indicating a strong attraction towards S6 within our cohort of HGG patients. Conversely, the transition from S6 to S5 was less likely, with a probability of 0.20. Hence, S6 could be interpreted as the dominant state associated with our cohort of HGG patients.

Looking at the switching rates of the two subgroups of patients, we found that patients in S5 switched at a faster rate (mean switching rate = 0.1 Hz) compared to those in S6 (mean switching rate = 0.07 Hz). Drawing from control theory, which posits that systems with high entropy or information load exhibit increased switching^39^, we attributed this difference to a higher state complexity in S5 compared to S6.

Although previous studies using static FC analysis have provided valuable insights into the preoperative alterations of whole-brain functional networks in HGG patients^19,22,26^, our initial dynamic results indicate that the system does not settle into a single state, rather, it switches between states. This highlights the need for a dynamic FC approach to capture the multi-stability regime^40^ of the brain, which exhibits fluctuations even under pathological conditions.

Regarding the characterisation of brain states based on their functional connectivity matrices (i.e., describing the statistical relationships among ICs), we found a global decrease in the strength of connections in the two pathological states compared to all the healthy states, consistent with the findings of Derks et al. regarding static FC^19^. In addition, as reported in^41^, changes in the resting-state functional connectome could be attributed to a global attenuation of the rs-fMRI BOLD signal caused by brain tumours, specifically the neurovascular remodelling associated with the lesion. However, our gradient-based analysis demonstrated that this global decrease was not linked to alterations in the underlying structure, which remained consistent across states. However, in contrast to other studies^37,42,43^ our approach did not reveal a clear separation between sensorimotor and auditory regions on one end and transmodal regions on the other end in terms of the first gradient. This discrepancy could be explained by the fact that we did not use BOLD signals derived from predefined functional parcellation; instead, we employed ICs time courses, which represent average signals of the nodes comprising them.

The graph-based analysis provided insights into the states in terms of centrality and the level of segregation/integration of the networks within states. Despite a preserved structure of hubs and their central role across states, our cohort of patients exhibited reduced global and local efficiency as well as clustering coefficient in both pathological states. This indicates a diminished overall capacity for information transfer and integration among distributed RSNs. When comparing the two pathological states, we observed reduced segregation in S5 compared to S6, which supports our hypothesis that S5 may represent a more complex brain state with intricate information exchange among networks. This was further supported by the modularity analysis conducted at the network level, which revealed a distinct relationship between the DAN and the other networks. In S5, the DAN was entirely segregated, while in S6, a high level of integration between the DAN and other high-cognitive networks such as FPN, CCN, FRN and BG was detected. These networks were assigned to the same module: the red one in Fig. 5.

The DAN and the FPN are two crucial attention networks included in our high-resolution functional parcellation. The DAN is responsible for gathering associations that link appropriate stimuli and responses to a particular task^44^. It comprises the intraparietal sulcus and the frontal eye fields, areas that are active when attention is oriented in space^45^. The FPN instead, is involved in executive control, which encompasses the ability to act based on goals^46^. It is associated with complex problem solving and working memory^47^. Within this context, our modularity results align with the framework proposed by Dixon and colleagues^46^, who demonstrated that the FPN can be decomposed into two separate subsystems referred to as “FPN_A_” and “FPN_B_”. FPN_A_ is connected to the DMN and regulates introspective processes, while FPN_B_ is connected to the DAN and regulates visuospatial perceptual attention. FPN_A_ is more closely associated with introspective processes and emotions, while FPN_B_, by exerting top-down control over the DAN, ensures that attention remains focused on task-relevant perceptual information to facilitate interactions with the environment, such as tool usage. Our modularity results (Fig. 5) trace the behaviour of FPN_A_ and FPN_B_ in the healthy states S1 and S4, respectively. However, in pathological conditions, FPN_A_ was not found, and FPN_B_ was present only in S6. Therefore, we speculate that the overall decline in cognitive status observed in our patient cohort can be attributed to a dysregulation of introspective processes, specifically those related to complex social reasoning associated with FPN_A_. On the other hand, the presence of the well-defined FPN_B_ (represented by the red module in S6) might mitigate this dysfunction by enabling the shift of spatial attention to specific objects and locations in the surrounding environment.

The final part of our analyses aimed at investigating whether the stratification of patients based on their occupancy in S5 or S6 could be associated with lesion-related pathophysiological features or the cognitive status of the patients. Interestingly, we found that the occupancy in a specific state did not depend on either the extension or the location of the lesion. However, through multivariate analysis, we demonstrated a strong relationship between state occupancy and the cognitive status of the patients (refer to Fig. 7). The predictive performance was high for both S5 (R^2^ = 0.73) and S6 (R^2^ = 0.77). The analysis identified the same neuropsychological tests as predictors for both pathological states, which aligns with a previous study conducted by our group^10^, where we found that changes in multiple networks only predicted attention deficits (R^2^ = 0.64). The selected tests mainly focused on attentive functions. Overall, regarding S5, we found that the highest model weights were associated with neuropsychological tests related to pure simple attentive functions, such as the Trail Making Test—A and Digit Span—forward^48^. Additionally, this state exhibited a completely segregated DAN, indicating that this network operates efficiently to support optimal performance in basic attentive tasks like the Trail Making Test—A and Digit Span—forward. On the contrary, when predicting the occupancy of S6, the highest weights were associated with tests that assess more complex attentive functions requiring executive components and involving working memory (Trail Making Test—B, Corsi backward)^49^. In addition, S6 was the state where a functional module emerged, with the DAN fully integrated with components involved in executive control functions, such as the FPN, forming the FPN_B_ subsystem. This integration between the DAN and FPN could explain the better performance of patients, predominantly found in S6, in more complex attentive tests like the Trail Making Test—B and Corsi backward. In this light, we thus hypothesised that the “most pathological” state could be considered as S5, whereas S6 might represent a state less affected by the pathology, allowing for compensatory mechanisms to occur.

The ability of the proposed framework to distinguish between two different cohorts of patients and individually predict the occupancy of specific functional states with a limited number of cognitive tests emphasises the clinical relevance of employing dynamic approaches in the study of FC.

While HMM is not the only dynamic approach used in these types of studies, we also evaluated the performance of the most widely used technique: the sliding windows followed by clustering approach^28^. The results obtained from this alternative approach are provided in the Supplementary material. However, although capable of detecting an optimal number of six dynamic clusters, this latter approach was less sensitive in separating patients from controls.

Nevertheless, multimodal studies that combine information from diffusion MRI for structural analysis, PET for metabolic assessment, fMRI for functional evaluation, and molecular genetic features of tumours would provide a more comprehensive understanding of specific traits in patients.

This study is not exempt from limitations. Firstly, the sample size of our patient cohort is relatively small considering the heterogeneity of tumour locations. Conducting further evaluations with larger patient groups and focusing on longitudinal assessments will be crucial to define the role of dynamic functional connectivity in advancing our understanding of brain tumours patients and translating this approach into clinical practice. Additionally, it would be beneficial to apply the dynamic approach presented here to other types of brain tumour to better delineate unique connectivity features in patients with high-grade gliomas and differentiate them from other classes of brain tumours. In the present study we focused exclusively on high-grade glioma patients to ensure a homogenous sample due to the prevalence of this glioma class in our initial dataset. Secondly, the use of an external dataset of healthy subjects represents another limitation. Due to the unavailability of data acquired from healthy subjects using the same MR scanner and sequences, we used raw data from a publicly available dataset acquired with an MRI scanner of the same field strength and similar rs-fMRI sequences as our patient dataset. Furthermore, the data pre-processing was conducted in the same manner for both datasets, minimising the possibility of results being influenced by different datasets. Finally, a significant technical limitation of using an HMM framework is that the inclusion of new rs-fMRI acquisitions into the model requires the complete re-running of the processing pipeline based on HMM, posing a challenge for scalability.

In summary, we have characterised functional dynamics in high-grade glioma patients for the first time in the literature using Hidden Markov Models applied to rs-fMRI data. Our findings demonstrate a decrease, rather than a disruption, in these dynamics among patients. Furthermore, we have shown that HMM is a sensitive dynamic approach capable of distinguishing patients from controls based on occupancy in dynamic brain states, with controls predominantly occupying four states and patients occupying two states. Although these two pathological states were not associated with tumour site or extension, we ultimately found that the occupancy of specific pathological states predicted cognitive deficits related to attentive functions. This underscores the potential of dynamic FC assessed through Hidden Markov Models as a clinical biomarker toward improving personalised medicine treatments.

## Methods

### Participants

Thirty-three patients suffering from high-grade glioma have been recruited and acquired at the University Hospital of Padova from July 2017 to April 2021. This study was approved by the Ethics Committee of University Hospital of Padova (No. 2771P prot:0065859/12). The inclusion criteria were: (1) de novo glioma (recurrences excluded), (2) aged 18 years or older, (3) absence of other neurological or psychiatric disorders, (4) availability of presurgical neurocognitive battery and MRI acquisitions, which had to include pre- and post-contrast T1w sequences, T2w, FLAIR and rs-fMRI, (5) absence of macroscopic artefacts in MR images.

All procedures were in accordance with the ethical standards of the institutional research committee and with the 1964 Helsinki declaration plus later amendments. All participants provided informed, written consent in accordance with the local University Hospital Institutional Review Board. As healthy controls (HCs) we employed data of 33 subjects (12 female, mean age 59.6 ± 12.7y) of the publicly available MPI-Leipzig Mind-Brain-Body dataset^35^.

### Data acquisition

MRI data of patients were acquired on a 3 T Siemens Biograph mMR scanner equipped with a 16-channel head-neck coil. Anatomical imaging included T1-weighted (T1w) 3D magnetization-prepared rapid acquisition gradient echo (repetition time (TR) = 2400 ms, echo time (TE) = 3.24 ms, inversion time (TI) = 1000 ms, flip angle (FA) = 8°, field of view (FOV) = 256 × 256 mm^2^, voxel size = 1 × 1 × 1 mm^3^, acceleration factor iPAT = 2, acquisition time (TA) = 5:45 min) images acquired both before and after contrast agent injection, a 3D T2-weighted (T2w) image (TR = 3200 ms, TE = 535 ms, FOV = 256 × 256 mm^2^, voxel size = 1 × 1 × 1 mm^3^), a 3D fluid attenuation inversion recovery (FLAIR) (TR = 5000 ms, TE = 284 ms, TI = 1800 ms, FOV = 256 × 256 mm^2^, voxel size = 1 × 1 × 1 mm^3^, iPAT = 2, acquisition time (TA) = 5:47 min) image. In addition, functional imaging comprised rs-fMRI scans acquired with a T2*-weighted gradient-echo echo planar imaging (EPI) sequence (TR = 1260 ms, TE = 30 ms, FA = 68°, FOV = 204 × 204 mm^2^, voxel size = 3 × 3 × 3 mm^3^, iPAT = 0, multi-band acceleration factor (MBAccFactor) = 2, volumes = 750, TA = 16:03 min, phase encoding direction antero-posterior) and two spin echo-EPI acquisitions with reverse phase encoding (TR = 4200 ms, TE = 70 ms, FA = 90°, FOV = 204 × 204 mm^2^, voxel size = 3 × 3 × 3 mm^3^, TA = 8.4 s) for EPI distortion correction purposes.

The HCs’ dataset^35^ comprises rs-fMRI scans acquired on a 3 T Siemens Magnetom Verio scanner with a T2*-weighted gradient-echo EPI sequence (TR = 1400 ms, TE = 39.4 ms, FA = 69°, FOV = 202 × 202 mm^2^, voxel size = 2.3 × 2.3 × 2.3 mm^3^, iPAT = 0, MBAccFactor = 4, volumes = 657, TA = 16:03 min, phase encoding direction antero-posterior) and two spin echo-EPI acquisitions with reverse phase encoding (TR = 2200 ms, TE = 52 ms, FA = 90°, FOV = 202 × 202 mm^2^, voxel size = 2.3 × 2.3 × 2.3 mm^3^, TA = 29 s).

### Neuropsychological tests

A neuropsychological battery was administered to assess various cognitive domains. Specifically, it included the Oxford Cognitive Screen (OCS)^50^, as well as several subtests of the Esame Neuropsicologico Breve 2 (Trail-Making-Test, forms A and B, Phonemic fluency, Prose memory immediate and delay recall, and Interference memory test)^51^, Boston Naming Test (BNT, visual naming), forward and backward Digit span and Corsi block-tapping test (short-term and working memory), the Nine-hole peg test (manual dexterity for both right and left hand).

For each subject, the raw scores from the 24 tests were converted to z-scores according to standardised normative values.

### Preprocessing of MRI data

The anatomical images of each patient were linearly registered to the patient T1w image with ANTs tool. An expert neuroradiologist with more than five years of experience (M.A.) employed these images to manually segment the lesions through the ITK-SNAP software. Thus, for each patient we obtained a tumour mask including the tumour core (contrast agent enhancing and non-enhancing regions) and the necrosis and a lesion mask including the tumour core, the necrosis, and the oedema.

Imaging data of both patients and HCs underwent an analogous structural and functional pre-processing. Structural pre-processing consisted in bias field correction *(N4BiasFieldCorrection*^52^), skull-stripping (*Multi-Atlas Skull Stripping*^53^*),* tissue segmentation with the *unified segmentation tool*^54^ and diffeomorphic non-linear registration (as implemented in ANTs SyN algorithm) to the symmetric MNI 2009c atlas. In the patient group, the last step was performed excluding the lesion mask.

Pre-processing of rs-fMRI data of both patients and HCs is fully described in^10,30^. In brief, after standard pre-processing steps, group independent components (ICs) were exploited by a back-reconstruction algorithm^55^ to estimate subject-specific spatial maps and time series of each IC. For each patient/HC we obtained 45 ICs that were grouped into 10 RSNs: visual (VIS), sensorimotor (SMN), auditory (AUD), cingolo-opercularis (CON), dorsal-attention (DAN), fronto-parietal (FPN), default-mode (DMN), cognitive-control (CCN), frontal (FRN) and basal ganglia (BG). The following steps were performed on the time courses of the 45 ICs as additional denoising step: (1) despiking, applied with the icatb_despike_tc function of the GIFT toolbox^56^, (2) multiple regression of the 6 head motion parameters, their temporal derivatives, mean WM and mean CSF signals^57^, (3) high-pass filtering (cut-off frequency = 1/128 Hz). No global signal regression was applied. Before applying HMM, the HCs data were interpolated in the time grid of patients' data (TR = 1.26 s) and the last 20 volumes of the patients' scan were discarded.

In terms of head motion, quantified with the framewise displacement^58^, no statistically significant differences were found between patients and HCs (Wilcoxon’s rank sum test, p-value = 0.19): the average framewise displacement and its standard deviation was 0.16 ± 0.05 mm for patients and 0.16 ± 0.04 mm for HCs.

### Hidden Markov model—setup

The BOLD time courses of each IC (zero mean and unit standard deviation) were temporally concatenated across all patients as well as HCs. After temporal concatenation, the input matrix for the HMM consisted of 66 subjects × 45 ICs × 730 time points. The HMM was then fitted to the temporally concatenated time courses, exploiting the HMM-MAR toolbox^31^. A multivariate Gaussian distribution was adopted for the observational model and thus each inferred state was characterised in terms of IC’s mean BOLD activation ([M × 1] vector) and covariance between ICs ([M × M] matrix), where M denotes the number of ICs. The choice of the model order was performed as in^30^. In brief, after fitting the model with the number of states ranging from 2 to 15, different indices were evaluated: the free-energy and the average log-likelihood (avLL), as indices of goodness of the fit and the coefficients of variation (CVs) to quantify the precision of the estimates.

### Characterisation of brain states dynamics

After running the model, we obtained the probability of each state being active at each time point for each subject. Next, the Viterbi algorithm^59^ was applied to assign a specific state to each time point. This process allowed us to derive two temporal metrics that summarise the brain dynamics captured by the HMM: the switching rate (SR), which represents the frequency of transitions between different states, and the fractional occupancy (FO), which indicates the proportion of time that each subject spends in each state. These subject-specific metrics were then averaged across patients and HCs. Statistical testing of differences between the two groups was performed using a Wilcoxon’s rank sum test, followed by multiple comparison correction (False Discovery Rate-FDR, α = 0.05). Moreover, the maximum occupancy of each patient in a particular state was computed, which resulted in a binary assignment of each patient to a single state.

### Characterisation of dynamic connectivity patterns

Once the temporal features (i.e., SR and FO) of brain states were assessed, we investigated how the patterns of dynamic FC differ between patients and HCs.

Firstly, we perform a Wilcoxon’s rank sum test to examine differences among states in the values of Pearson’s correlation among ICs, focusing on the upper triangular part of each FC matrix.

Secondly, using a gradient analysis^37^, we examined the RSNs organisation within each state. Finally, we employed a graph-based analysis to quantify metrics of functional segregation, integration, and centrality of RSNs within each state. The following sections provide a detailed description of these latter two analyses.

#### Gradient analysis

Exploiting the BrainSpace toolbox^60^, we constructed the affinity matrix by applying a Gaussian kernel to the FC matrices of the 6 states. The Laplacian eigenmap reduction approach was then utilised to decompose this matrix into principal eigenvectors that represent the axes of largest variance. Additionally, we employed a Procrustes rotation to align the gradients of all states with the first gradient of the averaged FC, computed as the average of the 6 states FC matrices. To assess the similarity between the first gradient of each state, we calculated the cosine similarity for each pairwise comparison.

#### Graph-based analysis

Utilising the BCT toolbox^61^, a sparsity threshold of 80%^62^ was applied to each state’s FC matrix. From these thresholded matrices, we computed two centrality metrics (degree and betweenness centrality), two segregation metrics (weighted local efficiency and clustering coefficient), and one measure of integration (global efficiency). A Wilcoxon’s rank sum test followed by multiple comparison correction (FDR, α = .05) was performed to identify significant differences in graph metrics between states. Finally, using Louvain's community detection algorithm^63^ within the framework described in^30^, we determined the modular organisation of RSNs by applying it to the thresholded FC matrices of each state. We then computed the Jaccard index to evaluate the similarity of modular organisation between states.

### Stratification of patients in states

After assigning each patient to a state based on their maximum FO, we investigated whether this stratification could be associated with (1) physiological features related to the lesion (lesion location or extension) and (2) the cognitive status of the patients.

#### Relation between occupancy and pathophysiological features

Pearson’s correlation between a patient’s occupancy in a state and patient's lesion extension was computed. We also verified whether persistence in a particular state could be related to the location of the tumour in the right or left hemisphere. Finally, we generated glioma frequency maps for the two patient groups: those predominantly occupying S5 and those predominantly occupying S6. These maps were created by dividing the sum of weighted lesion masks by the total number of patients in each group on a voxelwise basis.

#### Relation between occupancy and cognitive performances

After obtaining the normalised scores of each test for each patient, we performed a univariate analysis (Wilcoxon’s rank sum test, α = .05) to identify potentially important features that could distinguish the two subgroups of patients. Subsequently, a multivariate analysis framework was employed to enhance the predictive capacity of the individual tests by considering their relationships. Two models were built: model 1 used normalised test scores as predictors and FO in S5 as dependent variable, while model 2 used FO in S6 as the dependent variable. Prior to fitting the models, we performed feature selection via hierarchical clustering to address collinearity, using the Silhouette criterion to cut the tree. The representative feature in a cluster was chosen on the basis of the maximal correlation between the feature and the FO in S5 or in S6. The resulting reduced set of variables was then used in independent multivariate stepwise linear regression analyses to predict the FO in S5 or S6. To assess model fit, we computed the squared Pearson’s correlation (R^2^) coefficient between the model predictions and the dependent variables. Additionally, we examined the weights (β) of each feature to determine which test had the greatest impact on FO prediction.

## Supplementary Information

Supplementary Information.

## Data Availability

The datasets analysed during the current study are available from the corresponding author on reasonable request.
