# Supplementary material for: The dynamic functional connectivity fingerprint of high-grade gliomas
Source: Sci Rep. 2023 Jun 27;13:10389. doi: 10.1038/s41598-023-37478-2 (PMC10300022; doi:10.1038/s41598-023-37478-2)
Supplement: Supplementary file 1 — Supplementary Information. [file 41598_2023_37478_MOESM1_ESM.pdf]

**The dynamic functional connectivity fingerprint of high-grade gliomas**

Manuela Moretto, Erica Silvestri, Silvia Facchini, Mariagiulia Anglani, Diego Cecchin, Maurizio Corbetta, Alessandra Bertoldo

**SUPPLEMENTARY MATERIAL**

**Contents**

**1. Participants**……………………………………………………………………………………………………………2

Supplementary Table 1 - Patient's demographical and clinical information………………………………2

Supplementary Figure 1 - Lesion frequency map ………………………………………………………………3

**2. Hidden Markov Model – setup**…………………………………………………………………………………4

Supplementary Table 2 ………………………………………………………………………………………………4

3. **Characterization of brain states dynamics**……………………………………………………………4

Supplementary Figure 2 – Transition probabilities……………………………………………………………5

**4. Characterization of dynamic connectivity patterns**……………………………………………………5

Supplementary Table 3 – Jaccard similarity values………………………………………………………………5

**5. Comparison between HMM and Sliding Windows**………………………………………………………5

Supplementary Figure 3 – FC matrices of states' centroids……………………………………………………7

Supplementary Figure 4 – FO in states' centroids…………………………………………………………………7

Supplementary Table 4 – Correlation between FC matrices of HMM and SW……………………………8

Supplementary Table 5 – Structural similarity between FC matrices of HMM and SW ………………8

Supplementary Figure 5 – Graph metrics……………………………………………………………………………9

**6. Supplementary References**……………………………………………………………………………………9

1. **Participants**
   The demographics and clinical data of the patients are reported in Table 1. Figure 1 shows the frequency maps of the tumour in the patient population.

| *PATIENT ID* | *AGE* | *GENDER* | *HISTOLOGY* | *IDH1* | *HEMI* | *LOBE* | *T+O VOLUME [cm$^3$]* | *T VOLUME [cm$^3$]* |
|---|---|---|---|---|---|---|---|---|
| **1** | 74 | M | Diffuse glioneuronal tumour | \ | L | T | 30 | 5 |
| **2** | 43 | M | Glioblastoma | WT | L | F | 81.1 | 0.4 |
| **3** | 69 | M | Glioblastoma | WT | L | T | 70.1 | 66.2 |
| **4** | 67 | F | Glioblastoma | WT | L | P | 19.4 | 6.7 |
| **5** | 36 | M | Glioblastoma | WT | B | F- CC | 128.9 | 51.4 |
| **6** | 58 | F | Glioblastoma | WT | R | F-insular +splenium CC + P-O | 76 | 73.9 |
| **7** | 83 | F | Glioblastoma epithelial | WT | L | F-P | 60.8 | 36.6 |
| **8** | 42 | M | Glioblastoma | mutant | R | F | 139.2 | 123 |
| **9** | 56 | M | Glioblastoma | mutant | L | F | 127 | 88.8 |
| **10** | 32 | F | Glioblastoma | WT | R | Thalamic | 78.3 | 53.3 |
| **11** | 64 | M | Glioblastoma | WT | B | F-insular + CC | 67.9 | 54 |
| **12** | 75 | F | Glioblastoma | WT | L | T | 80.4 | 8.1 |
| **13** | 68 | M | Glioblastoma | WT | B | F-T-insular+ cingulate cortex+ splenium CC | 134.2 | 127.5 |
| **14** | 48 | F | Glioblastoma | WT | R | T + optic tract | 56.4 | 53.4 |
| **15** | 64 | F | Glioblastoma | WT | R | F | 12.8 | 9.3 |
| **16** | 46 | F | Glioneuronal neoplasm | mutant | L | F-insular | 83.1 | 83.1 |
| **17** | 77 | M | Glioblastoma | WT | L | T | 103 | 85.4 |
| **18** | 57 | M | Glioblastoma | WT | L | O-T | 50.9 | 43.1 |
| **19** | 49 | F | Glioblastoma | WT | L | T | 36.8 | 34.5 |

| | | | | | | | | |
|---|---|---|---|---|---|---|---|---|
| **20** | 74 | F | Glioblastoma | WT | L | F | 16.4 | 3.4 |
| **21** | 56 | F | Glioblastoma | WT | L | T | 6.1 | 6.1 |
| **22** | 49 | M | Glioblastoma | WT | L | T | 13.1 | 7.8 |
| **23** | 57 | M | Glioblastoma | WT | R | F | 191.6 | 108.3 |
| **24** | 80 | M | Glioblastoma | WT | R | O-T | 17.7 | 17.3 |
| **25** | 54 | F | Glioblastoma | WT | L | T | 25.7 | 24.3 |
| **26** | 67 | M | Glioblastoma | WT | L | F | 49.3 | 48.1 |
| **27** | 64 | F | Glioblastoma | WT | R | T-P | 155.9 | 155.9 |
| **28** | 73 | M | Glioblastoma | WT | R | F | 122.3 | 44.7 |
| **29** | 57 | F | Glioblastoma | WT | R | F | 112.4 | 99.5 |
| **30** | 50 | M | Glioblastoma | N.A. | L | F | 95.1 | 83.3 |
| **31** | 64 | F | Glioblastoma | WT | R | F | 125.1 | 62.6 |
| **32** | 73 | M | N.A. | N.A. | B | Splenium CC | 37.3 | 36.9 |
| **33** | 70 | M | Glioblastoma | WT | L | T | 18.8 | 16.3 |

**Supplementary Table 1**: Single patient's demographical and clinical information. Abbreviations: Hemi=involved hemisphere, T+O volume=extent of the tumour + oedema segmentation, T volume=extent of the tumour segmentation, CC= corpus callosum, F=frontal lobe, L=left, N.A.= not available, O=occipital lobe, P=parietal lobe, R=right, T=temporal lobe, WT=wild type, \=not measured)

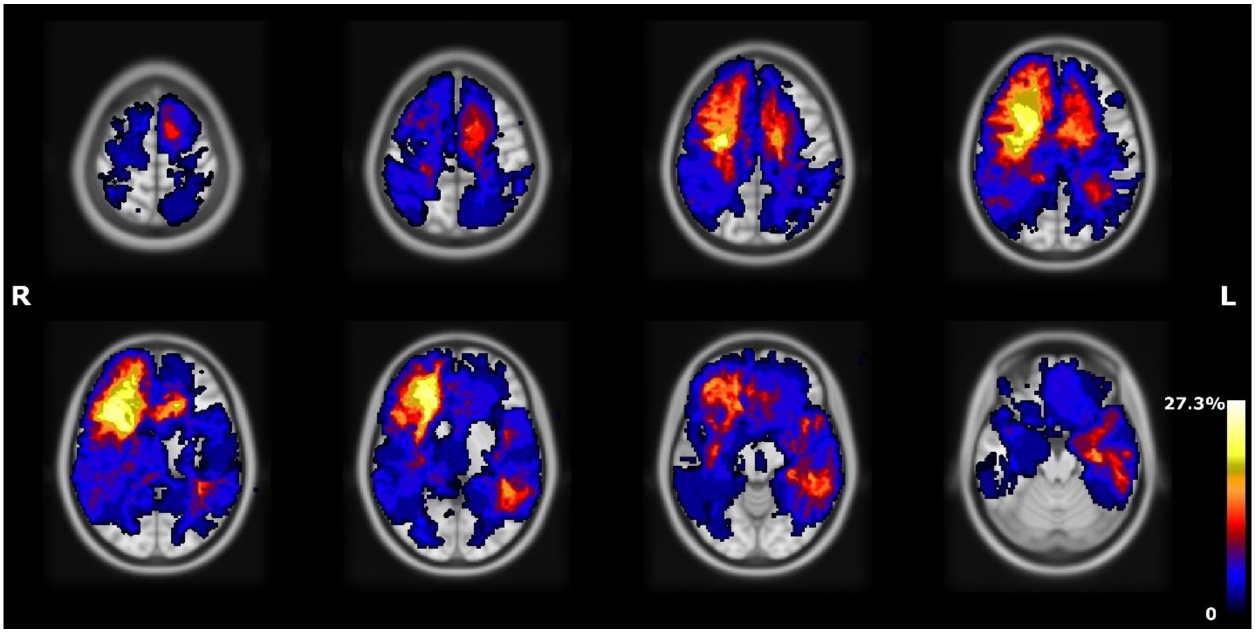


**Supplementary Figure 1:** Lesion frequency map across patients. Maps are over imposed to the MNI atlas (grey scale). Radiological convention.

2. **Hidden Markov Model – setup**

The model was fitted for different model orders and, in particular, from 2 to 15 states. Table 2 reports the values of the indices evaluated for the choice of the optimal order (K). While the free-energy (FE) showed a decreasing trend as the number of states increased, the average log-likelihood (avLL) reached the maximum for K=4, followed by K=9,6. Considering only these three orders, the coefficients of variation (CVs) suggested K=6 followed by K=9 as the best. Furthermore, the CVs obtained within each state, were, on average, lower for K=6 compared to K=9. Therefore, the number of states was set to K=6.

| MODEL ORDER | FE | avLL | CVs |
|---|---|---|---|
| **2** | 2441226 | -11338 | 0.233 |
| **3** | 2425304 | -15648 | 0.235 |
| **4** | 2412830 | -16656 | 0.232 |
| **5** | 2404905 | -17920 | 0.229 |
| **6** | 2397402 | -17452 | 0.227 |
| **7** | 2390761 | -18779 | 0.223 |
| **8** | 2384638 | -19102 | 0.217 |
| **9** | 2378470 | -17115 | 0.228 |
| **10** | 2374248 | -18200 | 0.222 |
| **11** | 2368210 | -18440 | 0.226 |
| **12** | 2363159 | -18717 | 0.223 |
| **13** | 2358134 | -17759 | 0.228 |

| | | | |
|---|---|---|---|
| **14** | 2353963 | -16652 | 0.219 |
| **15** | 2348874 | -17355 | 0.220 |

**Supplementary Table 2:** Three metrics, employed for the choice of the best HMM order, are reported: the free-energy (FE), the average log-likelihood (avLL), and the coefficients of variation (CVs).

## 3. Characterization of brain states dynamics

Figure 2 reports the transition probabilities among states, separately for patients and healthy controls.

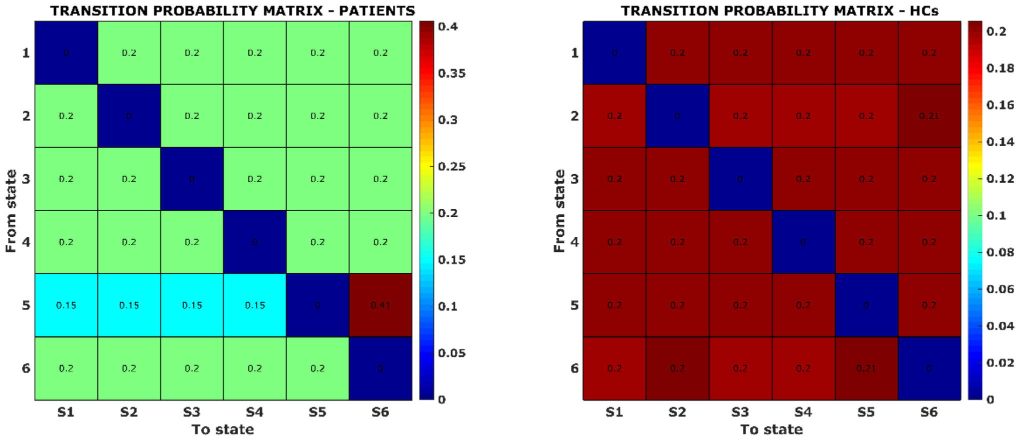


**Supplementary Figure 2:** Transition probabilities from one state (y-axis) to another (x-axis) for the two groups of healthy controls and patients separately.

## 4. Characterization of dynamic connectivity patterns

| *Jaccard* | *S1* | *S2* | *S3* | *S4* | *S5* | *S6* |
|---|---|---|---|---|---|---|
| ***S1*** | 1 | 0.45 | 0.56 | 0.53 | 0.54 | 0.61 |
| ***S2*** | - | 1 | 0.61 | 0.46 | 0.52 | 0.45 |
| ***S3*** | - | - | 1 | 0.43 | 0.63 | 0.50 |
| ***S4*** | - | - | - | 1 | 0.44 | 0.42 |
| ***S5*** | - | - | - | - | 1 | 0.77 |
| ***S6*** | - | - | - | - | - | 1 |

**Supplementary Table 2:** The table reports the Jaccard similarity values (Jaccard) between each state modular matrix. This index ranges between 0 and 1, where a similarity of 1 means that the states share the same modular organization.

## 5. Comparison between HMM and Sliding Windows

### *Methods*

To compare the results obtained through HMM with the gold-standard approach employed in the literature for dynamic connectivity analyses, we performed a sliding window followed by clustering analysis (SW)[1]. In details, we chose a window size of 73 TR (92 seconds) and a step size of 1 TR. Thus, for each subject we obtained 584 windowed FC matrices. For each windowed FC, only the values in the upper triangular part of the matrix were retained after z-Fisher transformation. Considering both the two groups of subjects, we thus obtained a matrix of dimensions 66 subjects × 584 windows × 990 correlation values. All subjects were then concatenated prior to performing the clustering analysis, thus obtaining a matrix of dimensions 38544×990.
Then, a K-means clustering was performed on the windowed FC matrices (Euclidean distance, 50 repetitions, number of clusters from 2 to 10). The Silhouette criterion[2] was employed for the choice of the optimal cluster size. For each cluster centroid the corresponding FC matrix was created and on the same analyses performed on the brain states FC matrices were used. In particular, the FO in each centroid was computed for each subject (FO_SW) and then to compare the FO_SW among the two groups, a Wilcoxon's rank sum test followed by multiple comparison correction ($\alpha$=0.05) was applied. The graph-based analyses and the same statistical tests described before were repeated on the centroids FC matrices.
To assess whether the two approaches (i.e., HMM and SW) could give comparable results in terms of connectivity and graph metrics associated to the states or clusters centroids, we matched each HMM state with a cluster centroid on the basis of the associated FC matrix. Specifically, the Pearson's correlation and the structural similarity index between the states FC matrices and the clusters centroids FC matrices were computed.

***Results***
The optimal cluster size resulted to be equal to six, employing the Silhouette criterion, thus six centroids FC matrices were characterized (Supplementary Figure 3).
When we compared the FO in different clusters between the two groups, we found statistically significant differences only in two clusters over six. Cluster 5 was mostly populated by patients, while cluster 6 by HCs (Supplementary Figure 4).
The match between FC matrices obtained with the two approaches (HMM and SW), evaluated through the Pearson's correlation and the structural similarity index (Supplementary Table 4 and 5), pointed out that the pathological state S5 could be clearly associated to cluster 5, while the healthy state S2 and S3 to cluster 2 and 3. For the remaining states, only a weak association was registered. Interestingly, in cluster 5, similar to the pathological S5, a statistically significant decrease in local efficiency and clustering coefficient, but not in the degree and betweenness centrality, was found (Supplementary Figure 5). Thus, while through the HMM analysis we were able to find two pathological brain states, through the SW analysis we could only find one pathological cluster, which probably encapsulated the connectivity features of both S5 and S6. Thus, in contrast to HMM-based analysis, this approach was less sensitive in separating patients from controls.

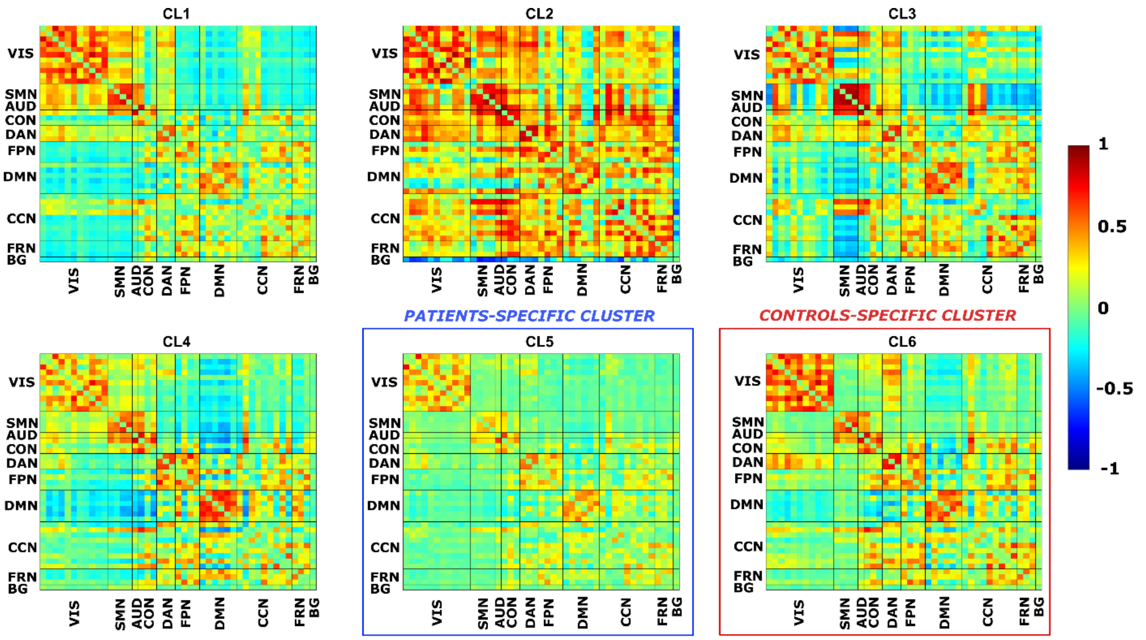


**Supplementary Figure 3:** Each panel represents the FC matrix associated to a particular cluster (CL1, CL2, etc.). In both the x- and y-axis, we have the 45 ICs, divided in the 10 functional domains. Warm colors represent high positive correlations values between ICs, whereas cool colors represent high anti-correlations values. We grouped together clusters centroids mostly populated by HCs (red box) and by patients (blue box).

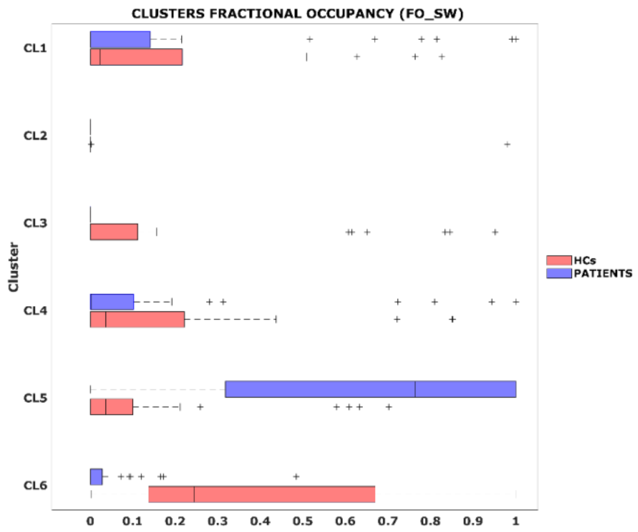


**Supplementary Figure 4:** The figure reports the distribution in different clusters of fractional occupancy, in blue for patients and in red for HCs. The bottom and top edges of each box indicate the 25th and 75th percentiles. The outliers are represented by the “+” symbol. In each boxplot, the solid line indicates the median.

| ***Corr*** | ***S1*** | ***S2*** | ***S3*** | ***S4*** | ***S5*** | ***S6*** |
|---|---|---|---|---|---|---|
| ***CL1*** | 0.58 | 0.58 | 0.59 | 0.59 | 0.61 | **0.62** |
| ***CL2*** | 0.55 | **0.66** | 0.62 | 0.60 | 0.60 | 0.58 |
| ***CL3*** | 0.54 | **0.61** | 0.58 | **0.61** | **0.61** | 0.60 |
| ***CL4*** | 0.49 | 0.48 | **0.54** | 0.44 | 0.52 | 0.53 |
| ***CL5*** | 0.63 | 0.61 | 0.67 | 0.57 | **0.72** | 0.68 |
| ***CL6*** | 0.71 | 0.68 | **0.74** | 0.65 | 0.73 | 0.70 |

**Supplementary Table 4:** The table reports the correlation (Corr) values between each FC matrix obtained through HMM and each FC matrix obtained through sliding windows. The highest values for each comparison are highlighted in bold.

| ***Ssim*** | ***S1*** | ***S2*** | ***S3*** | ***S4*** | ***S5*** | ***S6*** |
|---|---|---|---|---|---|---|
| ***CL1*** | 0.36 | 0.43 | 0.37 | **0.45** | 0.33 | 0.34 |
| ***CL2*** | 0.15 | **0.30** | 0.25 | 0.17 | 0.13 | 0.08 |
| ***CL3*** | 0.29 | **0.53** | 0.28 | 0.44 | 0.24 | 0.20 |
| ***CL4*** | **0.47** | 0.39 | 0.36 | 0.32 | 0.25 | 0.38 |
| ***CL5*** | 0.30 | 0.29 | 0.36 | 0.24 | **0.41** | 0.38 |
| ***CL6*** | 0.44 | 0.40 | **0.52** | 0.28 | 0.25 | 0.26 |

**Supplementary Table 5:** The table reports the structural similarity (Ssim) values between each FC matrix obtained through HMM and each FC matrix obtained through sliding windows. The highest values for each comparison are highlighted in bold.

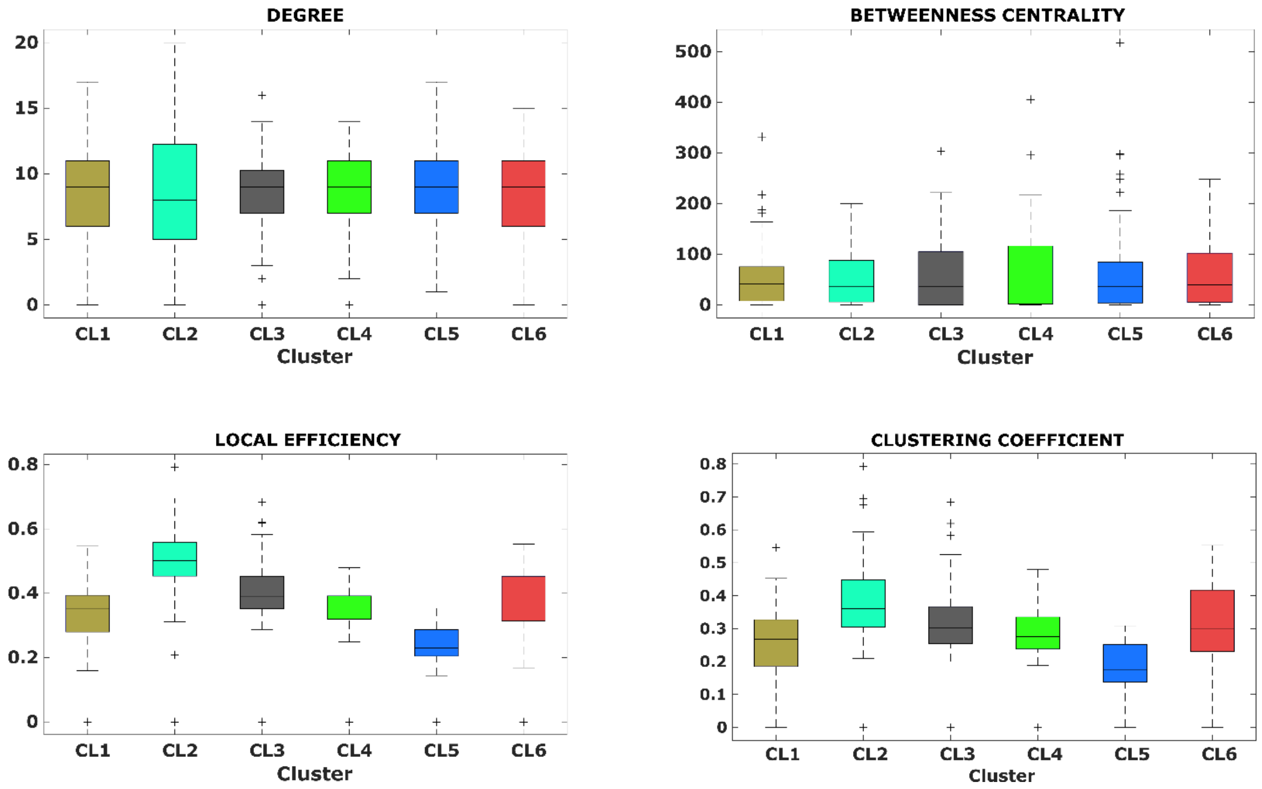


**Supplementary Figure 5:** The two top panels report the distribution of the two metrics of centrality (degree and betweenness centrality), in the form of boxplot, evaluated on the sparsified FC matrices of each cluster centroid (x-axis). In the bottom panels, the distribution of the two metrics of networks segregation (local efficiency and clustering coefficient) are reported.

## 6. Supplementary References

1. Allen, E. A. *et al.* Tracking Whole-Brain Connectivity Dynamics in the Resting State. *Cerebral Cortex* **24**, 663–676 (2014).
2. Rousseeuw, P. J. Silhouettes: A graphical aid to the interpretation and validation of cluster analysis. *Journal of Computational and Applied Mathematics* **20**, 53–65 (1987).
